# Supplementary material for: Racial and ethnic differences in foveal avascular zone in diabetic and nondiabetic eyes revealed by optical coherence tomography angiography
Source: PLoS One. 2021 Oct 22;16(10):e0258848. doi: 10.1371/journal.pone.0258848 (PMC8535464; doi:10.1371/journal.pone.0258848)
Supplement: Attachment — Submitted filename: Response to Reviewers.docx [file pone.0258848.s002.docx]

Dear Editorial Board,

We are very grateful to for these reviewers’ in-depth and thoughtful comments and suggestions on this paper. We have incorporated the suggested changes. Please find a point-by-point response below to all suggestions/comments provided by these reviewers.

| Review Comment | Author Response |
| --- | --- |
| Please ensure that your manuscript meets PLOS ONE's style requirements, including those for file naming. | Updated formatting. |
| We note that you received funding from a commercial source: That Man May See, Inc.  Please provide an amended Competing Interests Statement that explicitly states this commercial funder, along with any other relevant declarations relating to employment, consultancy, patents, products in development, marketed products, etc.  Within this Competing Interests Statement, please confirm that this does not alter your adherence to all PLOS ONE policies on sharing data and materials by including the following statement: "This does not alter our adherence to PLOS ONE policies on sharing data and materials.” (as detailed online in our guide for authors http://journals.plos.org/plosone/s/competing-interests). If there are restrictions on sharing of data and/or materials, please state these. Please note that we cannot proceed with consideration of your article until this information has been declared.  Please include your amended Competing Interests Statement within your cover letter. We will change the online submission form on your behalf. | That Man May See, Inc. is not a commercial organization. That Man May See, Inc., is a 501(c)(3) public charity that supports the Department of Ophthalmology and the Francis I. Proctor Foundation at the University of California, San Francisco. |
| Please include your full ethics statement in the ‘Methods’ section of your manuscript file. In your statement, please include the full name of the IRB or ethics committee who approved or waived your study, as well as whether or not you obtained informed written or verbal consent. If consent was waived for your study, please include this information in your statement as well. | Included in lines 246-247. |
| Please include captions for your Supporting Information files at the end of your manuscript, and update any in-text citations to match accordingly. | Added in lines 811-812. |
| Please review your reference list to ensure that it is complete and correct. If you have cited papers that have been retracted, please include the rationale for doing so in the manuscript text, or remove these references and replace them with relevant current references. Any changes to the reference list should be mentioned in the rebuttal letter that accompanies your revised manuscript. If you need to cite a retracted article, indicate the article’s retracted status in the References list and also include a citation and full reference for the retraction notice. | Checked. |
| Line 65: add reference or connect the 2 sentences | Sentence was deleted. |
| Lines 80-81: In fact, in the study of Chun et al (PMID: 31596848) differences in macular capillary parameters between healthy black and white subjects were assessed using OCTA. | Reference was added to line 189-191. |
| Lines 103: In the recent study of Shokr et al (PMID: 33576186), it was suggested that dry eye disease is associated with retinal microvasculature dysfunction. Even though the methods for assessing retinal microvasculature are different, it would be interesting to account for this factor as well either to only the nondiabetic group or also to diabetics if data available. | This sounds like a great next step and an interesting suggestion. However, we didn’t collect dry eye status or symptoms routinely in all the patients – we only noted dry eye disease if the patient reported it unsolicited. Thus, we wouldn’t be able to do that analysis retrospectively. Reference to this was added in the Discussion section in line 681-682. |
| What about myopia? Are they any differences among groups? OCTA studies have shown that there are significant microvascular retinal alterations in highly myopic eyes (e.g. PMID: 27820633). | Added myopia status based on AL to Table 1. Added line to methods about how this was calculated (281-282). There were no convincing differences in myopia prevalence between the race/ethnicity groups. |
| Was adding only the right eye based on any previous studies or not? You could still randomly select right-left. | Changed analyses to be linear regressions using both eyes and adjusting standard errors for patients with two eyes included. |
| Lines 138-140: Please, give more information about the DR screening program reading center. | Updated to: DR severity was graded from the color fundus photos by the department's DR screening program reading center, in which patients with diabetes undergo ultra-widefield fundus photography, and then the photographs are graded in standardized fashion by trained and supervised graders (Lines 291-294). |
| Lines 140-142: Which grading system did you use? What about proliferative DR? | PDR patients were excluded due to the small number of patients in this group. Mention of this was added (Line 297). A reference to the Scottish Grading Protocol was also added (Line 291). |
| Lines 142-144: You could move this sentence to the next paragraph where you are mentioning the statistical softwares and the tests that you used. | Deleted line. |
| It seems like that even though you collected data from 280 patients, since you applied one-way ANOVA you ended up analyzing data of almost half of the initial cohort. Is there a specific reason why you used this approach instead of using multilevel models also for the analysis of OCTA parameters which is actually the main purpose of your study? | Analyses were updated to using linear regressions utilizing all eyes with standard error adjustment for patients with two eyes included in the study. |
| Additionally, you mention that 280 patients were included but in table 2 you analyzed data of 271 patients (one eye). If so, you will need to update the demographic table in order to reflect the data presented in table 1 and also either change the number of patients included or explain what happened to those 9 patients whose data haven’t been analyzed. | Removed references to 280 and updated to 271. |
| In the discussion section, I would present less details of the studies that you mention and add/comment on some of the above. | Added mention of myopia (Lines 514-515) and dry eye associations (Lines 681-682) to the discussion section and removed numeric specifics of Poon et. al from discussion section. |
| Lines 140-142: It should be mentioned whether any severe NPDR or PDR cases occurred during the recruitment of diabetic patients. Were these cases excluded from the study analysis and why? | Addressed from Reviewer 1. |
| Lines 161-162: It is mentioned that “447 eyes from 280 patients were included in the study”, whereas in Table 1 the total number of included eyes and patients is n=432 and n=271, respectively. The authors should clarify the exact number of included subjects. | Addressed from Reviewer 1. |
| Furthermore, the authors had better revise the provided results in table 1 regarding “Years Since Diabetes Diagnosis”. More specifically, they should add whether the data are presented as mean (SD) and also explain what the subcategory “missing” exactly indicates. | “Data are presented as mean (SD) for continuous measures, and n (%) for categorical measures;” in table footer. Added: “Missing= datapoint missing from patient electronic health record” |
| Lines 193-202: Whether the provided data arose after adjusting for age, gender, hypertension and HbA1c should additionally be reported, since there is not a corresponding table to clarify this point. | Added: “There results were adjusted for age, gender, hypertension, and HbA1c where appropriate” (Line 499-500). |
| Lines 217: “..while NH black patients had the largest FAZ area.”: It would be helpful, if the authors reported the group to which this outcome refers, i.e. the mild to moderate NPDR group, since among non-diabetic subjects, according to table 3, Hispanic and Asian patients had larger FAZ area than both NH white and NH black people. | Added: “in patients with DR” (Lines 517-518). |
| Lines 51-52, 217-218, 262-263, 308-309: Only a few of the reported differences have been found to be statistically significant. More specifically, racial and ethnic differences in FAZ area among patients with diabetes without retinopathy did not show statistical significance. The difference in FAZ area between NH black and NH white participants at baseline was not statistically significant either. These points should be rendered clear while presenting the results. The conclusions of the study should also be drawn appropriately based on the data presented. | Language was added specifying group (Lines 154, 460-461, 496-497) and NH Black was removed from Lines 262-263 of original manuscript. Groups with similar magnitudes were emphasized, not just statistical significance. |
| Lines 237-238: The authors should explain in more detail the mentioned correlation and agreement of their findings with those of Poon et al., regarding both RNFL thickness and MDB thickness and area, in order to make this correlation sound and clear enough for the reader, since different retinal parameters are investigated in the studies. | Added: “The difference in population makeup between these studies highlights the importance of adequate statistical power for drawing conclusions about meaningful differences between groups. Depending upon the geographic location of a study center and its attendant patient population demographics, certain groups may be relatively over- or under-represented. In this instance, different retinal parameters were analyzed in Poon et al’s report versus the current study, making it difficult to compare the studies directly, but taken together the studies support the notion that racial and ethnic differences can contribute to reproducible differences in retinal anatomy that could have implications for disease susceptibility or progression” (Lines 549-557). |
| Lines 270-271 (Figure 1): It is advisable that the authors add the magnification of the pictures. The magnification should be similar, so that the pictures are comparable. | Added: “Images were taken unmodified for magnification as they were provided by the machine, then cropped the peripheral macula, placed the different eyes in this figure next to each other, locked the aspect ratio in the figure, and then scaled the figure to fit the page” (Lines 265-268). |
| Lines 60-61: It is advisable that “retinal microvasculature” are included as keywords. | Added to keywords. |
| Lines 127-130: Some of the parameters were automatically calculated. The authors should also explain how the other OCTA parameters were calculated or add the relating reference. | Added with reference: “Processing of these scans and calculations of parameters has been described previously” (Lines 276-277). |
| Lines 134-136: The authors should make a reference in the methods section of the manuscript to the final four race/ethnicity groups that are used in the study analyses. | Added reference to NCHS website (Line 285). |
| Line 138: The explanation of the abbreviation "MAR" in logMAR should be included, since it has not been mentioned previously in the manuscript. | Added reference (Line 290). |

Sincerely,

Jay M. Stewart, MD

Professor, Ophthalmology

University of California, San Francisco
